# The broad-host-range plasmid pSFA231 isolated from petroleum-contaminated sediment represents a new member of the PromA plasmid family

**DOI:** 10.3389/fmicb.2014.00777

**Published:** 2015-01-12

**Authors:** Xiaobin Li, Eva M. Top, Yafei Wang, Celeste J. Brown, Fei Yao, Shan Yang, Yong Jiang, Hui Li

**Affiliations:** ^1^State Key Laboratory of Forest and Soil Ecology, Institute of Applied Ecology, Chinese Academy of SciencesShenyang, China; ^2^College of Resources and Environment, University of Chinese Academy of SciencesBeijing, China; ^3^Department of Biological Sciences, Institute for Bioinformatics and Evolutionary Studies, University of IdahoMoscow, ID, USA

**Keywords:** broad-host-range plasmid, PromA plasmid family, complete sequence, plasmid backbone regions, comparative genomic analysis

## Abstract

A self-transmissible broad-host-range (BHR) plasmid pSFA231 was isolated from petroleum-contaminated sediment in Shen-fu wastewater irrigation zone, China, using the triparental mating exogenous plasmid capture method. Based on its complete sequence the plasmid has a size of 41.5 kb and codes for 50 putative open reading frames (orfs), 29 of which represent genes involved in replication, partitioning and transfer functions of the plasmid. Phylogenetic analysis grouped pSFA231 into the newly defined PromA plasmid family, which currently includes five members. Further comparative genomic analysis shows that pSFA231 shares the common backbone regions with the other PromA plasmids, i.e., genes involved in replication, maintenance and control, and conjugative transfer. Nevertheless, phylogenetic divergence was found in specific gene products. We propose to divide the PromA group into two subgroups, PromA-α (pMRAD02, pSB102) and PromA-β (pMOL98, pIPO2T, pSFA231, pTer331), based on the splits network analysis of the RepA protein. Interestingly, a cluster of hypothetical orfs located between *parA* and *traA* of pSFA231 shows high similarity with the corresponding regions on pMOL98, pIPO2T, and pTer331, suggesting these hypothetical orfs may represent “essential” plasmid backbone genes for the PromA-β subgroup. Alternatively, they may also be accessory genes that were first acquired and then stayed as the plasmid diverged. Our study increases the available collection of complete genome sequences of BHR plasmids, and since pSFA231 is the only characterized PromA plasmid from China, our findings also enhance our understanding of the genetic diversity of this plasmid group in different parts of the world.

## Introduction

Plasmids are extra-chromosomal self-replicating DNA elements within the microorganisms (Mela et al., 2008). They are important members of the mobile gene pool, and are among the most important contributors to horizontal gene transfer between bacteria (Frost et al., 2005). The broad-host-range (BHR) plasmids have been defined as those plasmids that can self-transfer themselves and can stably replicate and maintain in bacterial species from at least two subgroups within the Proteobacteria (e.g., between α- and β- Proteobacteria) (Szpirer et al., 1999; Sen et al., 2011). The BHR plasmids typically have mosaic genomes including two distinct regions (Thomas, 2000). The “plasmid backbone” genes encode proteins involved in the replication, maintenance, control and conjugative transfer of the BHR plasmid. Other plasmid regions are comprised of various “accessory” genes conferring important benefits to the host, including resistance to antibiotics (Rhodes et al., 2004), resistance to heavy metals (Schneiker et al., 2001), catabolic functions (Ono et al., 2007), and virulence determinants (Schlüter et al., 2008), etc.

Conjugative gene transfer mediated by BHR plasmids is generally believed to be a common and widespread mechanism for the transfer of genes across a broad phylogenetic range of bacteria (Top and Springael, 2003; Van der Auwera et al., 2009), and plays a crucial role in the adaptation of bacteria to environmental challenges and spread of antibiotic resistance (Jechalke et al., 2013). Despite the general agreement on the importance of BHR plasmids in the adaptive evolution of bacteria, the BHR plasmids being identified and completely sequenced are still few, initially limited by the high sequencing cost of first generation (Sanger) sequencing technology. To the best of our knowledge, no more than 15 BHR plasmids had been fully sequenced until 2006. With the development of next-generation sequencing methods, such as 454 pyrosequencing and Illumina high-throughput sequencing technology, more complete sequences of BHR plasmids have been added to the data pool in recent years, most of which were identified as IncP-1 plasmids (Sen et al., 2012; Brown et al., 2013). In spite of the increasing number of BHR plasmids identified, complete sequences of BHR plasmids available in GenBank are still not sufficient for systematically analyzing their genetic diversity. Thus, isolation and characterization of new BHR plasmids from environmental samples is still required to better understand the nature and evolutionary history of these important mobile genetic elements and their role in horizontal gene transfer.

Among the fully sequenced BHR plasmids, most are classified as the well-known incompatibility groups, such as IncP-1 (41 plasmids), IncW (5 plasmids), and IncU (4 plasmids), based on the backbone genes (Fernández-López et al., 2006; Norberg et al., 2011; Sen et al., 2011; Brown et al., 2013). Recently, a novel plasmid group, which could not be classified by the conventional inc/rep grouping system, was proposed by Van der Auwera et al. (2009) in terms of phylogenetic analysis of the complete genome sequence. This new BHR plasmid group was defined as PromA family, now including five members, namely plasmids pMOL98 (Gstalder et al., 2003), pIPO2 (Tauch et al., 2002), pSB102 (Schneiker et al., 2001), pTer331 (Mela et al., 2008), and pMRAD02 (Ito and Iizuka, 1971). Their RepA protein formed a separate cluster related to but distinct from the IncW plasmids, while several plasmid maintenance genes were most closely related to those of other incompatibility group, and the plasmid mating pair formation genes appeared similar to chromosomally encoded *Brucella* sp. *virB* genes (Schneiker et al., 2001; Tauch et al., 2002). Additionally, a putative PromA plasmid, defined as pMBUI6, was recently identified from freshwater sample (Brown et al., 2013). Important PromA-like features identified on pMBUI6 is the presence of topoisomerase gene (*topA*), the relaxase gene (*mobB*), and the long direct repeats in intergenic regions. Nevertheless, some of its backbone genes bear close similarity to pXF51 from *Xylella fastidiosa* but more distantly from PromA group.

In our present work, a self-transmissible BHR plasmid pSFA231 was isolated from petroleum-contaminated sediment in Shen-fu, China, by using the triparental mating method, selecting only for the plasmid's ability to mobilize a non-selftransmissible plasmid. Complete sequencing and phylogenetic analyses of this newly isolated plasmid revealed that it fell within the recently defined PromA plasmid group. Isolation of PromA plasmids with highly similar backbone sequences from different parts of the world provides further evidence for global spread of bacteria or their plasmids, and improves our understanding of the evolution of the PromA plasmid group.

## Materials and methods

### Site description and sampling

The sampling site was located in Shen-fu wastewater irrigation zone (123°35′ E, 41°44′ N), the largest petroleum wastewater irrigation zone in Northeast of China. A 70-km irrigation channel was constructed in 1960's, and the wastewater mainly comes from an oil refinery. After 50-year exposure to petroleum-containing wastewater, soils in the irrigation zone have been seriously contaminated (Li et al., 2005; Zhou et al., 2012). Plasmid pSFA231 was isolated from a sediment sample collected from down-stream of the irrigation channel. Surface sediment sample (0~10 cm) was collected with a shovel as described by Cook et al. (2001). The collected sediment sample was placed in a plastic bag, and then was transported to the laboratory on ice. The fresh sediment sample was kept at 4°C for plasmid isolation, and was air-dried and sieved (2 mm) for the analysis of the basic physical and chemical properties. The total petroleum hydrocarbons (TPH) was determined as 760.1 mg/kg using gravimetric method (Villalobos et al., 2008), and the pH value (soil: water = 1: 5) was 6.5. The organic matter, total nitrogen and available nitrogen of the sample were 12.8 g/kg, 0.7 g/kg, 4.0 mg/kg, respectively.

### Strains, plasmids, and media

A list of the strains and plasmids used in this study is provided in Table 1. Luria-Bertani (LB) broth was generally used to culture the strains. Solid medium was prepared by addition of 1.7% agar. When necessary for selection, antibiotics were added to the medium at the following concentrations: rifampicin, 50 μg/ml; gentamicin, 10 μg/ml; kanamycin, 50 μg/ml; streptomycin, 50 μg/ml. Mueller–Hinton (Becton, Dickinson, and Co., Franklin Lakes, NJ) agar was used for detection of antibiotic resistance. Cycloheximide (300 μg/ml) was added to LB agar (LBA) to prevent growth of fungus during screening of transconjugants.

**Table 1:** **Strains and plasmids used in this study**.

**Strains or plasmids**	**Genotype and relevant phenotype**	**References**
**STRAINS (CLASS)**		
*E. coli* (γ-Proteobacteria)		
MG1685 (K12 Rif)	Rif^R^ mutant of MG1655	Fox et al., 2008
JM109	*endA1 recA1 gyrA96 thi hsdR17* (r^−^_K_ m^+^_K_) *relA1 supE44Δ* (*lac-proAB*) [F′ *traD36 proAB laqI^q^Z ΔM15*]	Yanischperron et al., 1985
DH5α	Nal^R^	Sambrook et al., 1989
S17-1	Sm^R^	Mazodier et al., 1989
EC100	Sm^R^	Hale et al., 1984
*Agrobacterium tumefaciens* C58 (α-Proteobacteria)	Rif^R^	Wood et al., 2001
*Cupriavidus necator* JMP 228 (β-Proteobacteria)	Rif^R^	Top et al., 1995
**PLASMIDS**		
pBBR1MCS-5	BHR mobilizable cloning vector; Gm^R^	Yanischperron et al., 1985
pB10	Mercury-resistance	Schlüter et al., 2003
pUTminiTn*5*Km1	pUT replicon carrying miniTn*5*::Km1	De Lorenzo et al., 1990

### Exogenous isolation of the plasmid

Triparental exogenous isolation of plasmids was performed as described by Hill et al. (1992). A rifampicin-resistant strain *E. coli* MG1685 (K12 Rif^R^) (Fox et al., 2008) was used as the recipient to capture the plasmid from the sediment sample. *E. coli* JM109 (pBBR1MCS-5) (Yanischperron et al., 1985) was used as the donor, with the mobilizable plasmid pBBR1MCS-5 conveying resistance to gentamicin. Cultures of recipient and donor cells were grown overnight in LB broth containing corresponding antibiotics at 37°C.

Five grams of air-dried and sieved sediment sample was shaken for 1 h in 45 ml of sterile saline. The previously reported BHR plasmid pB10 (Schlüter et al., 2003) was added as a positive control. In the positive control flask, 5 g of soil sample was mixed with 100 μl of a 10^−1^ dilution (in saline) of a fully grown *E. coli* DH5α (pB10) culture (approximately 10^7^ CFU/g soil). The suspension was allowed to settle for approximately 30 min. The supernatant was transported to the Eppendorf tube and then centrifuged (4°C, 10,000 rpm) for 10 min. After centrifugation, the supernatant was discarded and 3 ml of LB solid medium was added to resuspend the pellet. Individually, 500 μl of donor, recipient, and sediment were dispensed into 1.5 ml Eppendorf tubes as controls for the mating. For every mating, 500 μl portions of overnight grown cultures of the donor and the recipient were mixed in an Eppendorf tube with 500 μl of sediment supernatant. All the mating and control preparations were centrifuged (4°C, 10,000 rpm) for 5 min, and the pellet was resuspended in 50 μl of LB broth. Then 30 μl of this cell suspension was spotted onto an LB agar plate. After overnight of mating at 30°C, using a sterile loop a portion of the biomass (~1/4–1/3 of the mating “spot”) from each droplet was resuspended in 500 μl of saline, and then agitated vigorously with a Vortex mixer. The cell suspension was serially diluted in saline, and 0.1 ml samples were plated on LB agar supplemented with rifampicin (50 μg/ml) and gentamicin (10 μg/ml). Transconjugant colonies were picked up after 2 days incubation and purified on the same agar medium. Finally, cultures of transconjugant cells were grown overnight in LB medium containing rifampicin (50 μg/ml) and gentamicin (10 μg/ml) at 37°C. Physical evidence that mobilizing plasmids were present was obtained by plasmid extraction by using the alkaline lysis method, followed by agarose gel electrophoresis.

### Detection of antibiotic resistance

To test antibiotic resistance of the isolated BHR plasmid, 1.8 ml overnight culture was centrifuged at 10,000 rpm for 10 min, and the cell pellets were washed with sterile saline for three times. The cells were resuspended in 500 μl sterile saline (CFU approximately 10^8^), and 250 μl bacterial suspension was added into 150 ml pre-heated Mueller–Hinton agar, mixed by inversion and quickly poured to make an inoculated plate. Paper discs (6 mm in diameter) containing different antibiotics of known amounts (Oxoid Ltd) were placed on the inoculated plates with sterile forceps. The type and content of antibiotics are the following: kanamycin (K30, 30 μg/slice), chloramphenicol (C30), Ciprofloxacin (CIP5), erythromycin (E15), amoxicillin (AMC30), rifampicin (RD5), macrodantin(F300), nalidixic acid (NA30), imipenem (IPM10), gentamycin (CN10), carbenicillin (CAR100), sulfamethoxazole (W5), ceftazidime (CAZ30), polymyxin B (PB300), miramycin (SH100), tetracycline (TE30). After 24-h incubation at 37°C, antibiotic resistance was determined by measuring the inhibition zones around the antibiotic paper discs compared with those of the recipient strain *E. coli* MG1685 and the donor plasmid pBBR1MCS-5. When the diameter of the inhibition zone was 8 mm, the transconjugant strain was considered as resistant to the antibiotic.

### Tagging the plasmid with mini-Tn*5* transposon

Antibiotic resistance test revealed that plasmid pSFA231showed no resistance to any antibiotics. To facilitate selection in further analysis, pSFA231 was tagged with a miniTn*5*::Km1 transposon (De Lorenzo et al., 1990) using a biparental mating/ mobilization strategy. *E. coli* MG1685 (pSFA231) served as the recipient and the transposon pUTminiTn*5*::Km1 was provided by donor strain *E. coli* S17-1. Transconjugants were picked and streaked on LB agar supplemented with kanamycin (50 μg/ml). The tagged plasmid (Km^R^) was eventually transferred to *E. coli* EC100 (Sm^R^) in a second round of biparental mating, at which point the captured plasmid was separated from the donor plasmid pBBR1MCS-5.

### Host range test

To determine the self-transferability and the host range, plasmid pSFA231 was transferred from *E. coli* EC100 (Sm^R^) to bacterial species from the other two Proteobacteria subgroups. Rifampicin-resistant (Rif^R^) strains, *Agrobacterium tumefaciens* C58 (α-Proteobacteria) (Wood et al., 2001) and *Cupriavidus necator* JMP228 (β-Proteobacteria) (Top et al., 1995), were used as recipients in biparental matings, respectively (Heuer et al., 2012). The plasmids were considered to transfer successfully if colonies could grow on medium added with kanamycin and rifampicin. Finally, plasmid was extracted by alkaline lysis method (Feliciello and Chinali, 1993) to confirm the presence of the plasmid in transconjugants.

### Sequencing and annotation

Plasmid DNA for sequencing analyses was prepared using the QIAGEN Plasmid Midi Kit (QIAGEN GmbH, Germany) according to the protocols provided by manufacturer. The whole genome of plasmid pSFA231 was sequenced by Illumina Hiseq 2000 high-throughput sequencing platform at the Majorbio Bioinformatics Technology Co. Ltd (Shanghai, China). The paired-end library was generated for high-throughput sequencing. Sequence assembly was primarily done with the SOAPdenovo (http://soap.genomics.org.cn/, version: v1.05) and GapCloser software (Li et al., 2010). To acquire the complete sequence of plasmid pSFA231, gaps in the plasmid sequence were closed using the general PCR method. Gene prediction was done using the Glimmer 3.0 (http://www.cbcb.umd.edu/software/glimmer). The annotation information of the predicted genes was obtained through blastp alignment between the amino acid sequences of the predicted genes and the Nr database information using BLAST 2.2.24+. The annotated nucleotide sequence of plasmid pSFA231 was submitted to the GenBank database under the accession number KJ850907.

### Bioinformatic analyses

GC contents were calculated using the BioXM software. GenBank was searched for similar sequences using BLAST (Altschul et al., 1997). The PromA plasmids used in the comparative analysis are listed in Table 2. Due to the unclear classification, PromA-like plasmid pMBUI6 was not included in the comparison. Nucleotide sequences and amino acid (AA) sequences translated from the coding sequences were aligned using ClustalX (Thompson et al., 1997), then Mega 6 (Tamura et al., 2013) was used to infer the phylogenetic trees using the neighbor joining algorithm with the best-fit model. The SplitsTree program was used to infer the phylogenetic network (Huson and Bryant, 2006). The Pairwise genetic distance based on each backbone protein was calculated by Mega 6 using the Jones–Taylor–Thornton method (Norberg et al., 2011). A circular plasmid map was generated using the SnapGene Viewer (http://www.snapgene.com/products/snapgene_viewer). Schematic diagrams of multiple alignments of plasmids were produced by manually realigning the linear plasmid maps generated by the SnapGene Viewer. The identity scores of translated DNA sequences were calculated by the BLAST program, bl2seq (Tatusova and Madden, 1999).

**Table 2:** **General features of the PromA plasmids family**.

**Plasmids**	**Length (bp)**	**Sources**	**Accession no**.	**References**
pMOL98	55,563	Hydrocarbon-polluted soil in Essen, Germany	FJ666348	Van der Auwera et al., 2009
pIPO2T	45,319	Wheat rhizosphere in Wageningen, the Netherlands	AJ297913.2	Tauch et al., 2002
pSB102	55,578	Rhizosphere of alfalfa in Braunschweig, Germany	NC_003122.1	Schneiker et al., 2001
pTer331	40,457	Dune soil on Wadden Island Terschelling, the Netherlands	NC_010332.1	Mela et al., 2008
pMRAD02	47,003	Strain *Methylobacterium radiotolerans* JCM2831 isolated from the rice in Japan	NC_010509.1	Unpublished (only available at Genbank)

## Results

### Isolation and general characterization of the plasmid pSFA231

A BHR plasmid, named here as pSFA231, was isolated from the petroleum-contaminated sediment. Antibiotic resistance test indicated that plasmid pSFA231 carried no additional antibiotic resistance compared to the recipient strain and donor plasmid. For further analysis, the plasmid was marked by a mini-Tn*5* transposon carrying a kanamycin resistance gene cassette. The tagged plasmid was eventually transferred to *E. coli* EC100 (Sm^R^) by a second round of biparental mating.

To investigate the transferability of the exogenously isolated plasmid pSFA231, the selected clone of *E. coli* EC100 (pSFA231) was used as donors in biparental matings with rifampicin-resistant strains of *Agrobacterium tumefaciens* C58 (α-Proteobacteria) and *C. necator* JMP228 (β-Proteobacteria). Results showed that plasmid pSFA231 can self-transfer and replicate in representative strains from three different subgroups of Proteobacteria.

### Basic genome sequence information of the BHR plasmid pSFA231

**Figure 1:**
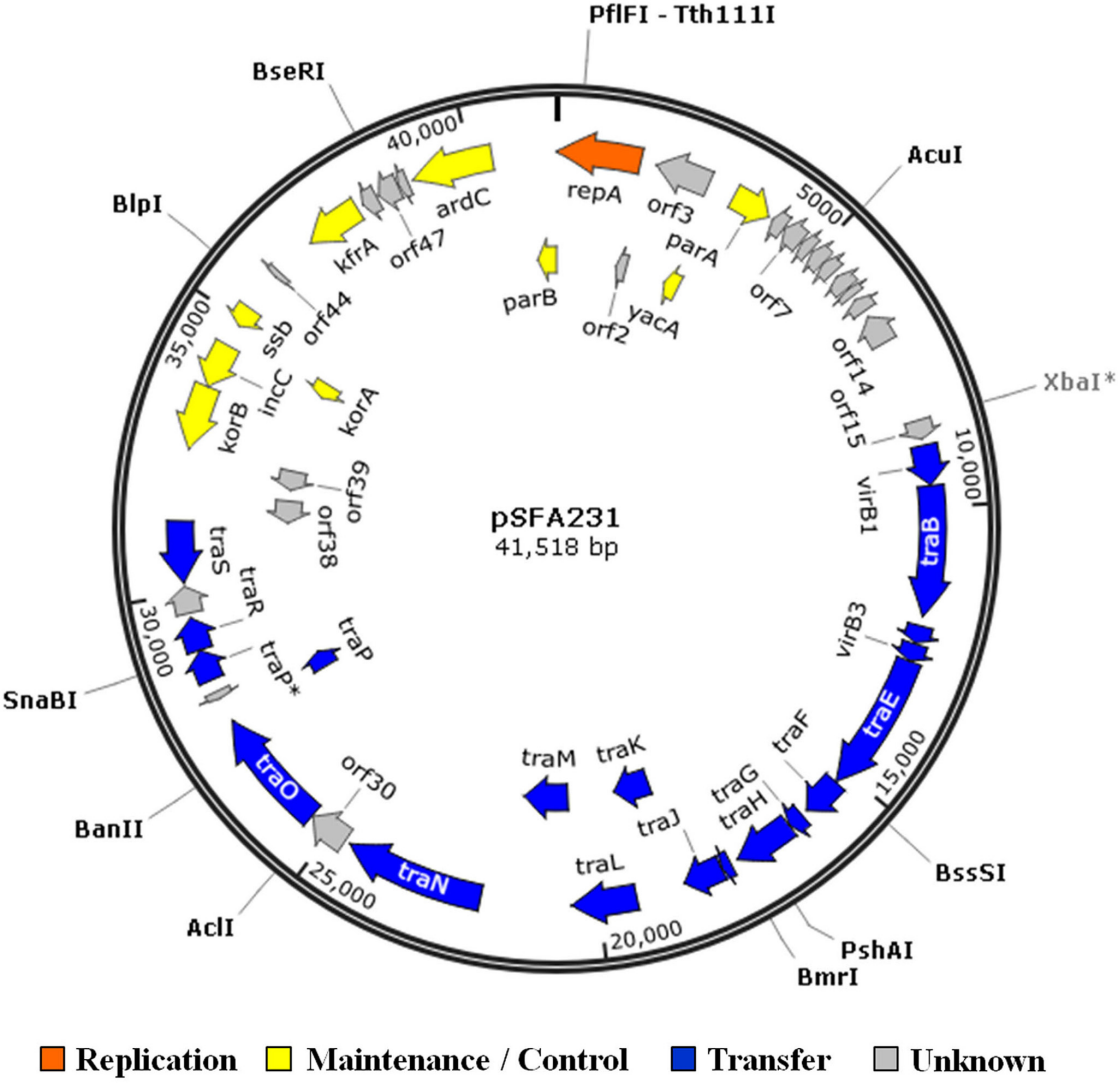
**Circular map of plasmid pSFA231**. The 50 orfs identified in the nucleotide sequence of pSFA231 are located on a circular map. The orfs are shown by arrows indicating the direction of transcription. Different colors indicate replication (orange) and partitioning functional regions (yellow), and the transfer functional regions (blue). Hypothetical coding regions are shown in gray. The genes transcribed in clockwise orientation are in the outer perimeter and those transcribed in anti-clockwise orientation are in the inner perimeter. The restriction enzyme cutting sites are shown as a filled circle.

The complete nucleotide sequence of the wild-type plasmid pSFA231 is determined to be 41518 bp with a GC content of 60.54%. Annotation of the sequence revealed a total of 50 orfs, of which 22 are transcribed on one strand and 28 on the other (Figure 1). The sequence has about 84% coding ratio with an average orf length of 705 bp. The closest relatives (with highest amino acid identity scores) of these orfs in GenBank are summarized in Table 3. Among the 50 predicted orfs, 29 were attributed to certain biological functions, 19 orfs coding for conserved hypothetical proteins, and the remaining 2 predicted genes do not have any known homologs. The putative known coding regions of pSFA231 are dominated by essential plasmid backbone genes involved in plasmid replication, maintenance and control, and conjugative transfer (Table 3, Figure 1).

**Table 3:** **Location of putative coding regions on plasmid pSFA231 and the closest relatives of the deduced proteins**.

**Gene**	**Positions**	**Function**	**Proteins with highest amino acid identity (%)**	**Accession no**.
*repA*	C 1–1497	Replication	RepA from pMOL98 (99%)	ACT97177.1
*orf2*	C 1472–1732	Unknown	MOL98_2 from pMOL98 (100%)	ACT97178.1
*orf3*	C 1768–2748	Unknown	MOL98_3 from pMOL98 (100%)	ACT97179.1
*yacA*	C 2723–3076	Partitioning	YacA from pTer331 (100%)	YP_001672069.1
*parA*	D 3250–3957	Partitioning	ParA from pTer331 (98%)	YP_001672068.1
*orf6*	C 4034–4273	Unknown	MOL98_18 from pMOL98 (99%)	ACT97193.1
*orf7*	C 4299–4676	Unknown	hypothetical protein from *Pseudomonas* sp. HPB0071 (60%)	WP_010799325.1
*orf8*	C 4687–4911	Unknown	hypothetical 8 kDa protein from uncultured bacterium (100%)	NP_663744.1
*orf9*	C 4930–5193	Unknown	ORF41 from pTer331 (100%)	YP_001672067.1
*orf10*	C 5202–5468	Unknown	ORF40 from pTer331 (100%)	YP_001672066.1
*orf11*	C 5541–5810	Unknown	hypothetical 8.5 kDa protein from uncultured bacterium (99%)	NP_663739.1
*orf12*	C 5834–5974	Unknown	ORF27 from pMOL98 (100%)	ACT97201.1
*orf13*	C 6062–6328	Unknown	ORF28 from pMOL98 (100%)	ACT97202.1
*orf14*	C 6537–6985	Unknown	ORF29 from pMOL98 (99%)	ACT97203.1
*orf15*	D 8465–8797	Unknown	ORF31 from pMOL98 (99%)	ACT97205.1
*virB1*	D 8912–9616	Transfer	VirB1 from pTer331 (98%)	YP_001672057.1
*traB*	D 9626–11941	Transfer	TraB from pMOL98 (99%)	ACT97207.1
*traC*	D 12,106–12,414	Transfer	TraC from pMOL98 (100%)	ACT97208.1
*virB3*	D 12,436–12,747	Transfer	VirB3 from pTer331 (100%)	YP_001672054.1
*traE*	D 12,754–15,234	Transfer	TraE from pMOL98 (100%)	ACT97210.1
*traF*	D 15,239–15,961	Transfer	TraF from pMOL98 (98%)	ACT97211.1
*traG*	D 16,065–16,361	Transfer	TraG from pMOL98 (98%)	ACT97212.1
*traH*	D 16,373–17,455	Transfer	TraH from pMOL98 (100%)	ACT97214.1
*traI*	D 17,592–17,756	Transfer	TraI from pMOL98 (100%)	ACT97215.1
*traJ*	D 17,762–18,472	Transfer	TraJ from pMOL98 (100%)	ACT97216.1
*traK*	D 18,469–19,341	Transfer	TraK from pMOL98 (100%)	ACT97217.1
*traL*	D 19,341–20,501	Transfer	TraL from pMOL98 (100%)	ACT97218.1
*traM*	D 20,485–21,552	Transfer	TraM from pMOL98 (99%)	ACT97219.1
*traN*	D 22,100–24,604	Transfer	TraN from pMOL98 (99%)	ACT97221.1
*orf30*	D 24,706–25,401	Unknown	ORF48 from pMOL98 (99%)	ACT97241.1
*traO*	D 25,413–27,605	Transfer	TraO from pMOL98 (99%)	ACT97222.1
*traP*	D 27,602–28,012	Transfer	TraP from pMOL98 (99%)	ACT97224.1
*orf33*	C 28,048–28,185	Unknown	–	–
*traQ*	D 28,404–28,964	Transfer	TraQ from pMOL98 (98%)	ACT97225.1
*traR*	D 28,978–29,556	Transfer	TraR from pMOL98 (100%)	ACT97226.1
*orf36*	D 29,645–30,124	Unknown	ORF55 from pMOL98 (99%)	ACT97228.1
*traS*	C 30,179–31,273	Transfer	TraS from pMOL98 (100%)	ACT97229.1
*orf38*	C 31,270–31,812	Unknown	ORF57 from pMOL98 (100%)	ACT97230.1
*orf39*	C 32,079–32,555	Unknown	ORF11 from pTer331 (99%)	YP_001672037.1
*korB*	C 32,552–33,697	Partioning	KorB from pTer331 (99%)	YP_001672036.1
*incC*	C 33,698–34,489	Partioning	IncC from pMOL98 (100%)	ACT97233.1
*korA*	C 34,486–34,863	Partioning	KorA from pMOL98 (100%)	ACT97234.1
*ssb*	C 34,888–35,247	Partioning	Ssb from pMOL98 (100%)	ACT97235.1
*orf44*	C 35,954–36,082	Unknown	–	–
*kfrA*	C 36,812–37,846	Partioning	KfrA from pMOL98 (99%)	ACT97236.1
*orf46*	C 37,949–38,239	Unknown	ORF64 from pMOL98 (100%)	ACT97237.1
*orf47*	C 38,267–38,659	Unknown	ORF65 from pMOL98 (100%)	ACT97238.1
*orf48*	C 38,663–38,845	Unknown	ORF4 from pTer331 (100%)	YP_001672030.1
*ardC*	C 38,927–40,387	Partioning	ArdC from pMOL98 (99%)	ACT97239.1
*parB*	C 41,055–41,518.1	Partioning	ParB from pMOL98 (98%)	ACT97240.1

Similarity searches showed that most of the predicted orfs in plasmid pSFA231 coding for proteins are highly similar to those from plasmid pMOL98, and the remaining orfs were annotated to the proteins from plasmid pTer331 (Table 3). Both pMOL98 and pTer331 are listed as the members of the recently defined PromA plasmid family (Van der Auwera et al., 2009), leading to the conclusion that plasmid pSFA231 is a member of the PromA group.

### Plasmid pSFA231 harbors a replication module unique to the PromA family

**Figure 2:**
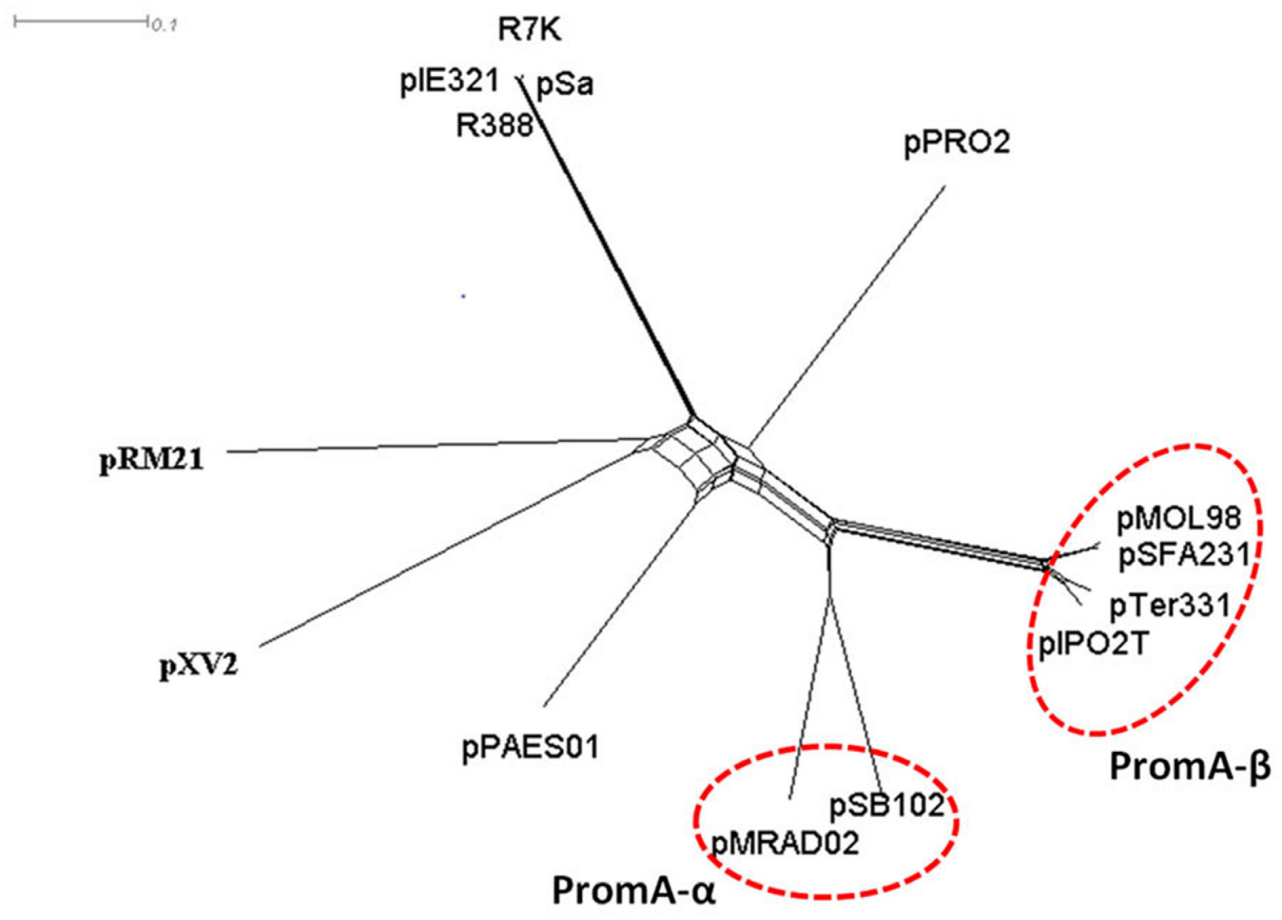
**Phylogenetic network of RepA proteins of selected IncW and PromA plasmids, using the neighbor joining algorithm on protein distances with Poisson correction**. Phylogenetic distance (amino acid difference percentage) was indicated by the length of the tree branches and the scale bars.

Before PromA plasmids were recommended as a new family, their RepA proteins were reported to show some degree of identity with the RepA proteins of IncW plasmids. To further reveal the phylogenetic relationship of the PromA plasmids with IncW plasmids, a splits network (Figure 2) was constructed for 1000 bootstrap replicates of the RepA protein phylogeny of the currently reported PromA members and the selected IncW plasmids, R388 [GenBank accession: BR000038], R7K [GenBank accession: NC_010643.1], pPAES01 [GenBank accession: CP001109], pSa [GenBank accession: U3071.1], pXV2 [GenBank accession: AF201825.1], pIE321 [GenBank accession: NC_010716.1], pRM21 [GenBank accession: NC_001755.1], and pPRO2 [GenBank accession: NC_008608] (Fernández-López et al., 2006). The network, which presents a combinatorial generalization of phylogenetic trees, presents a star-like topology with six main clades (Figure 2). It can be visualized that the six PromA plasmids formed a cluster distinct from IncW plasmids, and the PromA-clade was clearly divided into two sub-clades. Plasmids pMRAD02 and pSB102 were clustered separately from the other four members, and was suggested here as the PromA-α. The initiator protein RepA of plasmid pSFA231 had 99, 91, and 90% similarity to the corresponding protein of pMOL98, pTer331, and pIPO2T, respectively, and thus, they all together were proposed to be grouped into the PromA-β.

**Figure 3:**
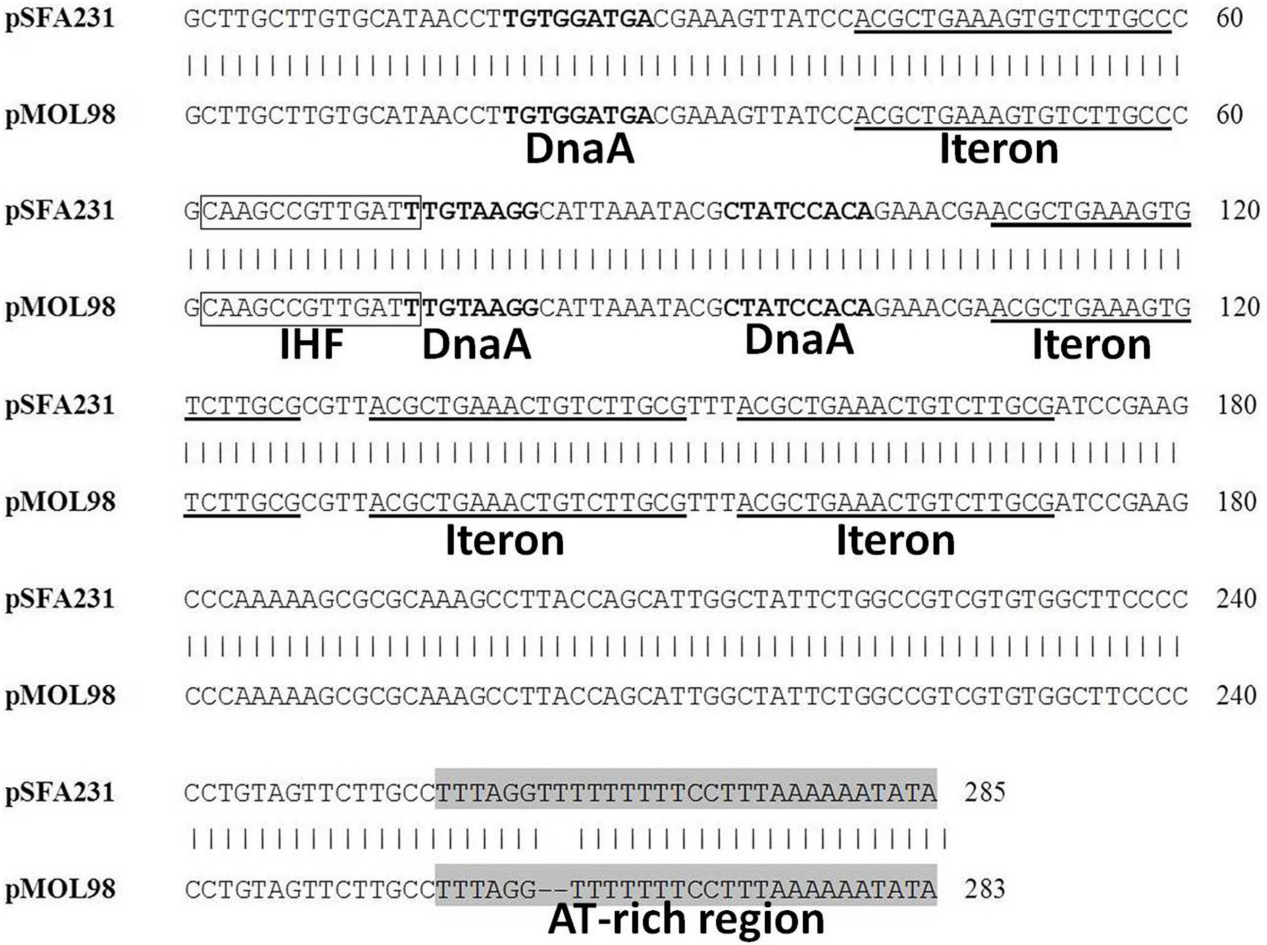
**Alignment of the pSFA231 replicative origin region with the putative oriV regions of pMOL98**. DnaA refers to DnaA boxes, IHF refers to putative Integration Host Factor binding sites.

Comparison of the oriV region of plasmid pSFA231 and its closest homolog, pMOL98, further verified that the replication module of pSFA231 was similar to PromA plasmids. Like other members of the PromA family, pSFA231 was characterized as θ-type mode of replication, with an oriV-like region being located at 5.1 kb downstream of the *repA* gene. Within this region, we identified four putative iterons (RepA binding site), which are identical to the iteron sequences from pMOL98. Furthermore, a putative DnaA-binding site, a potential integration host factor (IHF) site and an AT-rich (86.67%) region were also found in this region (Figure 3).

### Comparison of the backbone structure of PromA plasmids

**Figure 4:**
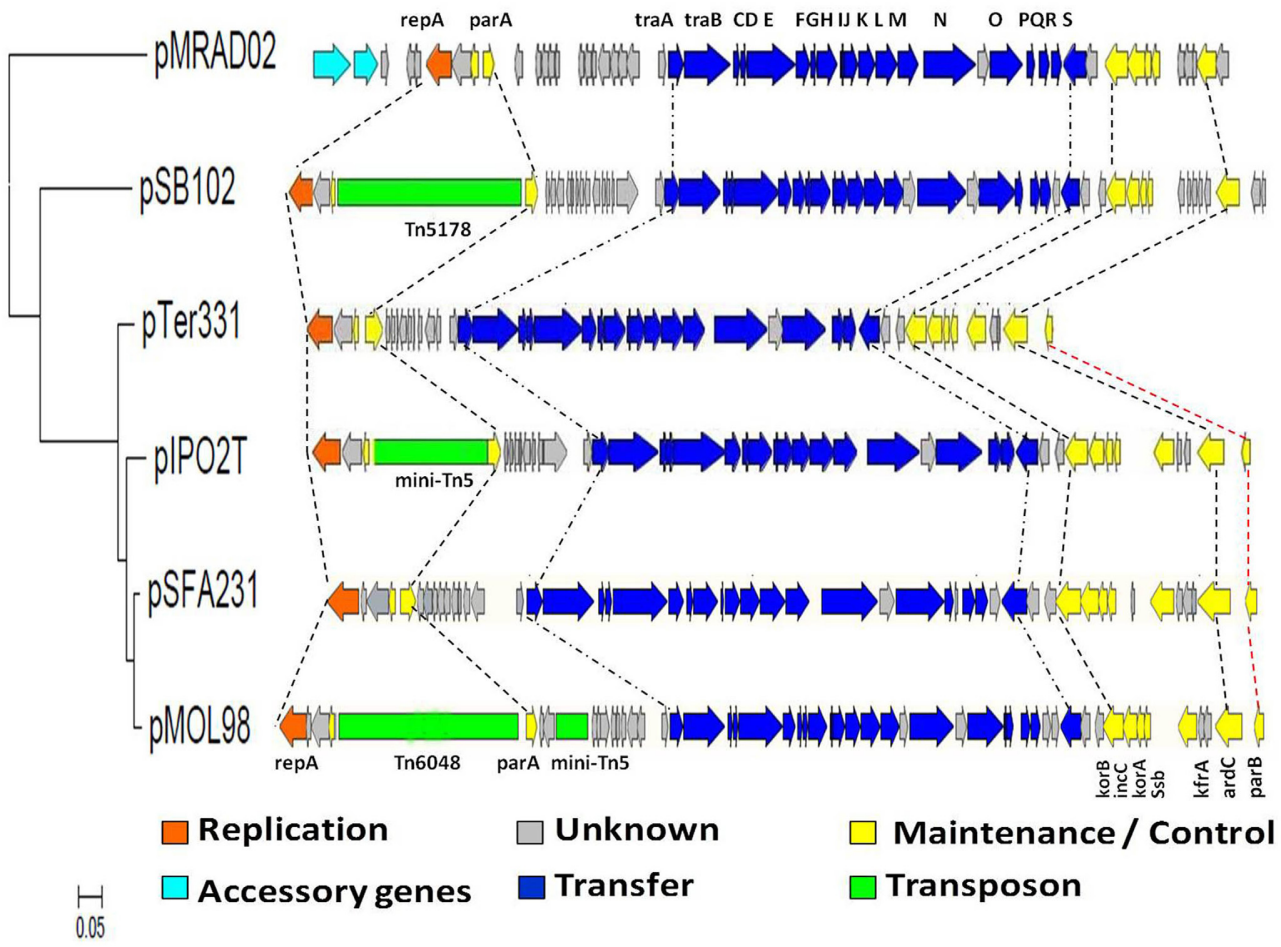
**Schematic diagram of linear alignment of the 6 PromA plasmids**. Phylogenetic tree was constructed based on the DNA sequences of concatenated 25 backbone genes using the Tamura- Nei model. The orfs are represented by block arrows. Predicted functions are indicated by the color key featured below the figure. Key backbone genes and accessory genes are annotated in the corresponding regions.

Comparison of the genome sequence between plasmid pSFA231 and the other five PromA plasmids, pMOL98, pIPO2, pTer331, pSB102, and pMRAD02, revealed a high level of structural similarity. They shared the common backbone regions including functional modules for replication (*repA*, oriV), conjugative transfer (*tra*), and maintenance/control (*yacA, parA, korB, incC, korA, ssb, kfrA, ardC*, and *parB*) (Figure 4). Nevertheless, phylogenetic divergences were found in specific loci indicating that PromA plasmids may have complex evolutionary histories. For instance, *traO* and traO^*^ present in pMOL98 provide evidence for duplication (Figure 4). The putative relaxase gene *traS* locus on pSFA231, pMOL98, pSB102, and pMRAD02 is not visible on the pTer331 and pIPO2T, additionally, the *krfA* locus and *parB* locus located on pSFA231, pMOL98, pIPO2T, and pTer331, are almost entirely unrecognizable on pSB102 and pMRAD02. The presence and absence of specific backbone genes are most likely the result of insertions and/or deletions.

To evaluate the evolutionary history of the backbone regions, the pairwise genetic distances among the PromA plasmids were calculated based on the amino acid (AA) sequences of each backbone protein using the Jones-Taylor-Thornton method (Table S1). The AA similarity differed across the 6 plasmids. Among all the 5 known PromA plasmids, pMOL98 had the closest genetic distance to pSFA231 with respect genes of *traC, virB3, traE, traH, traI, traJ, traK, traR, incC, korA, and ssb*. In contrast, almost all the pMRAD02 backbone proteins showed the greatest genetic distance to our newly isolated plasmid pSFA231, except the *traQ* and *traR*. As for the plasmid pTer331, genes *yacA, parA, virB1, traC, traM, traO, korB, and kfrA* presented the closest, while genes *traP, traQ, traR*, and *parB* presented the greatest genetic distance to pSFA231.

To further reconstruct their evolutionary history, the phylogenetic analysis of the whole backbone regions, which are conserved and present in all 6 plasmids, was performed. It was observed that the gene coding for protein TraS is absent in plasmids pTer331 and pIPO2T, and plasmids pSB102 and pMRAD02 are lacking in the genes coding for proteins KfrA and ParB (Table S1). Thus, the *traS* gene sequence and the DNA sequences from *kfrA* to *parB* were excluded from the alignment, leaving two large backbone regions. One region contains 21 continuous genes, organized from *repA* to *traR*, while the other region includes genes *korB, incC, korA*, and *ssb*. Although the amino acid sequences translated from the counterparts of backbone genes in selected PromA plasmids (gene *yacA* in pMOL98, pSB102, and pMRAD02; *parA* in pSB102; *traG* in pMRAD02; *traP* in pMRAD02; *korB* in pIPO2T; *incC* in pIPO2T; and *korA* in pTer331, pIPO2T, and pMRAD02) were defined as hypothetical proteins, the corresponding genes are present in these plasmids, allowing us to include them in our comparative genomic analysis. A phylogenetic tree constructed from the concatenated DNA sequence showed that pSFA231 is most similar to pMOL98, then to pIPO2T and pTer331, but is phylogenetically divergent from pSB102 and pMRAD02 (Figure 4).

We also performed a phylogenetic analysis using six concatenated backbone gene products (Figure S1), namely, RepA, TraB, TraE, TraN, TraO, and KorB. Not surprisingly, the results are consistent with those based on the entire backbone regions, indicating that to simplify the process of comparative genomic analysis we can choose gene products with relatively large size and high level of synteny as targets for comparison.

### The accessory regions and transposons of the PromA plasmids

One cluster of hypothetical orfs (*orf6*-*orf15*) with unknown functions was detected between *parA* and *traA* of the PromA members. Interestingly, we found that this gene cluster shows high DNA similarity with the corresponding regions on pMOL98 (97%), pIPO2T (95%), and pTer331 (95%), inferring that these genes (or some of them) may also be part of the common backbone of PromA-β sub-clade, although we still lack direct evidence. While transposons Tn*5178* and Tn*6048* were found to be inserted between *yacA* and *parA* gene on plasmids pSB102 and pMOL98, respectively. No transposon was detected in the corresponding region on our plasmid pSFA231, nor on pTer331.

## Discussion

In the present study, a new BHR plasmid pSFA231 was isolated from a sediment sample collected from a petroleum-wastewater irrigation channel, using the triparental exogenous plasmid capture method. It was recommended that choosing donor and recipient strains that are phylogenetically distinct will increase the possibility of obtaining plasmids with a broad host range (Top et al., 1994). In our analysis, we also tried the donor/recipient system of *E. coli* DH5α (pSU4814)/*C. necator* JMP228 (β-Proteobacteria, Rif^R^), but no self-transmissible BHR plasmids were isolated from the same sediment sample (data not shown). Although the plasmid pSFA231 was isolated by using a donor and recipient that both belonged to the same subgroup of Proteobacteria, further host range test showed that pSFA231 could successfully self-transfer and replicate in α-, β -, and γ-Proteobacteria.

Sediments are likely important reservoirs of BHR plasmid (Paul et al., 1991; Sobecky et al., 1997; Smalla et al., 2006). The rich biofilm structures in sediments (Westall and Rincé, 1994; Sutherland et al., 1998) may provide the bacteria more opportunities for cell contact and therefore transfer of mobile genetic elements. Moreover, BHR plasmids are frequently captured from contaminated environmental samples (Gstalder et al., 2003; Schlüter et al., 2007). BHR plasmids play an important role in the adaptation of bacterial populations to pollution stress, and long-term contamination may induce horizontal gene exchange and reshuffling of genetic information between phylogenetically distinct prokaryotes (Top and Springael, 2003). With long-term exposure to petroleum contamination, the Shen-fu wastewater irrigation zone likely provides a natural pool of BHR plasmids. Actually, a set of diverse BHR plasmids were captured from dozens of samples collected from Shen-fu irrigation zone in our experiment (unpublished data), including 4 IncP-1ε plasmids, 2 unknown plasmids, together with the PromA plasmid reported in this study. We expect an increasing number of phylogenetically diverse self-transmissible plasmids would be identified from this region by trying different capturing methods, such as biparental mating method or endogenous plasmid isolation (Top et al., 1994). Unexpectedly, in contrast to the BHR plasmids isolated from sludge collected from wastewater treatment plant (WWTP) (Schlüter et al., 2003, 2007; Wibberg et al., 2013), plasmid pSFA231 carried no antibiotic resistance genes. This phenomenon may not be abnormal. In a previous comparison of antibiotic resistance profiles of plasmids captured from non-polluted creek and WWTP effluent, no clear difference was found in the proportions of resistant plasmids captured from the two sites (Brown et al., 2013).

Based on a similarity search for putative orfs and subsequent comparative analysis, plasmid pSFA231 was proposed as a new member of the recently defined PromA plasmid family. Compared with the other incompatibility groups, PromA members were symbolized by a distinct replication initiation module, which contains a specific oriV-like region and a RepA protein (Gstalder et al., 2003; Van der Auwera et al., 2009). While the typical replication module of the IncP-1 group, into which most BHR plasmids have been classified, consists of *trfA* and *ssb* genes, and also an oriV region (Brown et al., 2013). Splits network analysis of the RepA protein clearly separated PromA members into two sub-divisions, illustrating the slight difference in their replication modules. Thus, in this study, we proposed to divide PromA family into two sub-groups, though there were only six members available. We believe that with more BHR plasmids being added into this recently defined group, new subgroups may be recommended in the future.

The phylogenetic information of backbone genes provides fundamental information on the “long-term” evolutionary history of BHR plasmids. In this study, the concatenated backbone regions of PromA family were compared between plasmid pSFA231 and five previously reported members. Although concatenation of backbone genes is problematic when there are distinct evolutionary histories of different functional regions of the plasmids (Sen et al., 2012), it is still a recommended method for inferring the evolutionary history of plasmids with higher backbone similarity (Norberg et al., 2011). Also, the SplitsTree algorithm allows us to discern the presence of divergent histories. Here, we use gene products for comparison rather than DNA sequence, because proteins are built from 20 amino acids while DNA only contains four different bases, meaning that the “signal-to-noise ratio” in protein sequence alignments is much better than in alignments of DNA (Wernersson, 2003). We found that all 6 PromA members share the highly conserved backbone regions, comprising replication, maintenance and control, and conjugative transfer functions. These plasmids were isolated from a variety of habitats, such as sediment, rhizosphere and soils, distributed in different locations in The Netherlands, Germany, Japan, and in this study, China (Table 2). It is of great interest that these geographically distinct BHR plasmids harbor backbone genes of high similarity. This fact suggests the wide distribution of PromA members.

Despite members of PromA family sharing a common backbone structure, phylogenetic analysis of the complete backbone regions still revealed significant divergence among the PromA members. Obviously, pSFA231 is more diverged from plasmids pMRAD02 and pSB102 than from pMOL98 and pTer331 (Figures 2, 4), which supports our recommendation on dividing the PromA family into two sub-divisions. During the process of evolution, the genetic organization of the backbone regions can rearrange via inversion, transposition, and duplication/loss processes (Price et al., 2001; Darling et al., 2004; Joshi et al., 2005), leading to the presence of diverse plasmids belonging to the same incompatibility group. For example, plasmid pMOL98 has two *traO* genes, providing evidence for duplication (Figure 4). In addition, lack of the *traS* genes in the backbone regions of the plasmids pSFA231, pMOL98, pSB102, and pMRAD02 indicated an indel during evolution.

Gains and losses of transposons and other MGEs often happen in BHR plasmid evolution (Kamachi et al., 2006). It was observed that transposons are embedded in the backbone of the plasmids pSB102 and pMOL98. The Tn*5178* located on plasmid pSB102 confers mercury resistance (Schneiker et al., 2001), and Tn*6048* carried on plasmid pMOL98 was characterized as multiple metal response (Van der Auwera et al., 2009). Because the lack of inserted elements is considered to be a sign of ancestry (Norberg et al., 2011), plasmid pSFA231 and pTer331 without any transposons are most likely to be closely related to the ancient ancestor of the PromA-β sub-clade.

A cluster of hypothetical orfs located between the *parA* and *traA* gene of plasmid pSFA231 catches our attention, due to its striking high similarity with the corresponding regions on pMOL98, pIPO2T, or pTer331. The conservation demonstrated by these hypothetical proteins indicates that these hypothetical ORFs may contain “essential” backbone proteins of PromA-β subgroup, although we still have no further direct evidence. Another hypothesis is that this orfs cluster is accessory genes that were first acquired and then stayed as the plasmid diverged. We made such a hypothesis just because this orfs cluster was exactly situated near the *parA* locus, which was usually considered as a “hot-spot” in PromA plasmids for insertion of accessory elements (Minakhina et al., 1999; Van der Auwera et al., 2009). The reason why the accessory genes insert into or near the region of *parA* locus so frequently is that this site contains a consensus AT-palindromic sequence and topology of the target DNA (Liu et al., 2005; Tobes and Pareja, 2006). Further analysis is required to confirm the structure and function of these hypothetical proteins in future studies.

In the present study, a new self-transmissible BHR plasmid pSFA231 was isolated from the petroleum-contaminated sediment and was recommended as a new member of the recently defined PromA family, based on phylogenetic and comparative genomic analysis. The present work is of great significance to add new information to the BHR plasmid sequence database that now exists. We believe that in this era of high-throughput sequencing, more members of BHR plasmids would be fully sequenced, and the extension of the database will improve our understanding of the genetic diversity of this important mobile genetic element.

### Conflict of interest statement

The authors declare that the research was conducted in the absence of any commercial or financial relationships that could be construed as a potential conflict of interest.
