# Supplementary material for: The broad-host-range plasmid pSFA231 isolated from petroleum-contaminated sediment represents a new member of the PromA plasmid family
Source: Front Microbiol. 2015 Jan 12;5:777. doi: 10.3389/fmicb.2014.00777 (PMC4290620; doi:10.3389/fmicb.2014.00777)
Supplement: Supplementary file 1 [file DataSheet1.DOCX]

**The broad-host-range plasmid pSFA231 isolated from petroleum-contaminated sediment represents a new member of the PromA plasmid family**

Xiaobin Li^1, 2^, Eva M. Top^3^, Yafei Wang^1^, Celeste J. Brown^3^, Fei Yao^1, 2^, Shan Yang^1^, Yong Jiang^1^, Hui Li^1*^

^1^State Key Laboratory of Forest and Soil Ecology, Institute of Applied Ecology, Chinese Academy of Sciences, Shenyang, China

^2^College of Resources and Environment, University of Chinese Academy of Sciences, Beijing, China

^3^Department of Biological Sciences, Institute for Bioinformatics and Evolutionary Studies (IBEST), University of Idaho, Moscow, ID, USA

***Correspondence:**

Dr. Hui Li

Chinese Academy of Sciences

Institute of Applied Ecology

State Key Laboratory of Forest and Soil Ecology

No. 72 Wenhua Road

Shenyang, 110164, China

E-mail: [huili@iae.ac.cn](mailto:huili@iae.ac.cn)

**Table S1 | Genetic distances between pSFA231 and other fully sequenced PromA plasmids.**

|  | **pMOL98** | **pTer331** | **pIPO2T** | **pSB102** | **pMRAD02** |
| --- | --- | --- | --- | --- | --- |
| RepA | 0.01 | 0.06 | 0.06 | 0.44 | 0.59 |
| YacA | * | 0.00 | 0.02 | * | * |
| ParA | 0.06 | 0.02 | 0.03 | * | 2.11 |
| TraA | 0.02 | 0.02 | 0.06 | 0.47 | 0.63 |
| TraB | 0.01 | 0.13 | 0.09 | 0.35 | 0.58 |
| TraC | 0.00 | 0.00 | 0.05 | 0.38 | 1.02 |
| TraD | 0.00 | 0.00 | 0.02 | 0.21 | 0.67 |
| TraE | 0.00 | 0.01 | 0.02 | 0.20 | 0.37 |
| TraF | 0.05 | 0.09 | 0.11 | 0.49 | 0.53 |
| TraG | 0.02 | 0.04 | 0.05 | 0.50 | * |
| TraH | 0.00 | 0.04 | 0.04 | 0.26 | 1.08 |
| TraI | 0.00 | 0.02 | 0.02 | 0.41 | 0.86 |
| TraJ | 0.00 | 0.00 | 0.02 | 0.36 | 0.55 |
| TraK | 0.00 | 0.02 | 0.01 | 0.43 | 0.43 |
| TraL | 0.01 | 0.02 | 0.06 | 0.40 | 0.50 |
| TraM | 0.01 | 0.01 | 0.01 | 0.29 | 0.42 |
| TraN | 0.01 | 0.16 | 0.03 | 0.40 | 0.68 |
| TraO | 0.02 | 0.02 | 0.04 | 0.47 | 1.02 |
| TraP | 0.05 | 3.35 | 3.32 | 0.64 | * |
| TraQ | 0.02 | 4.72 | 4.49 | 0.49 | 1.03 |
| TraR | 0.00 | 3.23 | 3.19 | 0.73 | 0.68 |
| TraS | 0.02 | - | - | 0.50 | 0.82 |
| KorB | 0.08 | 0.02 | * | 0.70 | 0.71 |
| IncC | 0.00 | 0.01 | * | 0.32 | 0.46 |
| KorA | 0.00 | * | * | 0.35 | * |
| Ssb | 0.00 | 0.01 | 0.02 | 0.32 | 0.62 |
| KfrA | 0.01 | 0.01 | 0.10 | - | - |
| ArdC | 0.01 | 0.04 | * | * | 0.50 |
| ParB | 0.02 | 0.03 | * | - | - |

Note：Genetic distances between the amino-acid sequence of each backbone gene in pSFA231 and the corresponding sequences in the other five analyzed plasmids. The plasmid(s) with the greatest distance to pSFA231 is marked in red and the plasmid(s) with the shortest distance is marked in blue for respective gene. Genes not present in specific plasmids are marked with “–” and genes that are defined as encoding hypothetical proteins in GenBank database are marked with “*”.


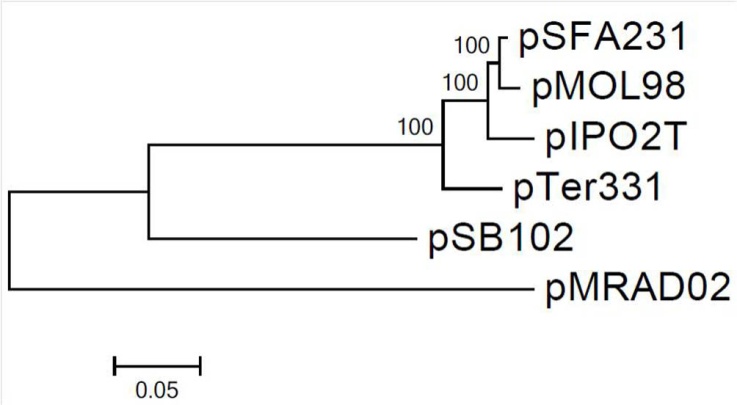


**FIGURE S1 | Phylogenetic tree of six shared backbone proteins (RepA, TraB, TraE, TraN, TraO, and KorB) using the neighbor joining algorithm on protein distances with Dayhoff model.**
